# Gut-derived GIP activates central Rap1 to impair neural leptin sensitivity during overnutrition

**DOI:** 10.1172/JCI126107

**Published:** 2019-08-12

**Authors:** Kentaro Kaneko, Yukiko Fu, Hsiao-Yun Lin, Elizabeth L. Cordonier, Qianxing Mo, Yong Gao, Ting Yao, Jacqueline Naylor, Victor Howard, Kenji Saito, Pingwen Xu, Siyu S. Chen, Miao-Hsueh Chen, Yong Xu, Kevin W. Williams, Peter Ravn, Makoto Fukuda

**Affiliations:** 1 Children’s Nutrition Research Center, Department of Pediatrics and; 2 Dan L. Duncan Cancer Center and Center for Cell Gene and Therapy, Baylor College of Medicine, Houston, Texas, USA.; 3 Division of Hypothalamic Research, Department of Internal Medicine, The University of Texas Southwestern Medical Center at Dallas, Dallas, Texas, USA.; 4 National Laboratory of Medical Molecular Biology, Institute of Basic Medical Sciences, Chinese Academy of Medical Sciences and Peking Union Medical College, Beijing, China.; 5 Department of Physiology and Pathophysiology, Xi’an Jiaotong University School of Medicine, Xi’an, Shaanxi, China.; 6 AstraZeneca, R&D BioPharmaceuticals Unit, Cardiovascular, Renal and Metabolism, Cambridge, United Kingdom.; 7 AstraZeneca, R&D BioPharmaceuticals Unit, Cardiovascular, Renal and Metabolism, Gaithersburg, Maryland, USA.; 8 Department of Molecular and Cellular Biology, Baylor College of Medicine, Houston, Texas, USA.; 9 AstraZeneca, R&D BioPharmaceuticals Unit, Department of Antibody Discovery and Protein Engineering, Cambridge, United Kingdom.

**Keywords:** Metabolism, Neuroscience, G-protein coupled receptors, Leptin, Obesity

## Abstract

Nutrient excess, a major driver of obesity, diminishes hypothalamic responses to exogenously administered leptin, a critical hormone of energy balance. Here, we aimed to identify a physiological signal that arises from excess caloric intake and negatively controls hypothalamic leptin action. We found that deficiency of the gastric inhibitory polypeptide receptor (*Gipr*) for the gut-derived incretin hormone GIP protected against diet-induced neural leptin resistance. Furthermore, a centrally administered antibody that neutralizes GIPR had remarkable antiobesity effects in diet-induced obese mice, including reduced body weight and adiposity, and a decreased hypothalamic level of SOCS3, an inhibitor of leptin actions. In contrast, centrally administered GIP diminished hypothalamic sensitivity to leptin and increased hypothalamic levels of *Socs3*. Finally, we show that GIP increased the active form of the small GTPase Rap1 in the brain and that its activation was required for the central actions of GIP. Altogether, our results identify GIPR/Rap1 signaling in the brain as a molecular pathway linking overnutrition to the control of neural leptin actions.

## Graphical abstract

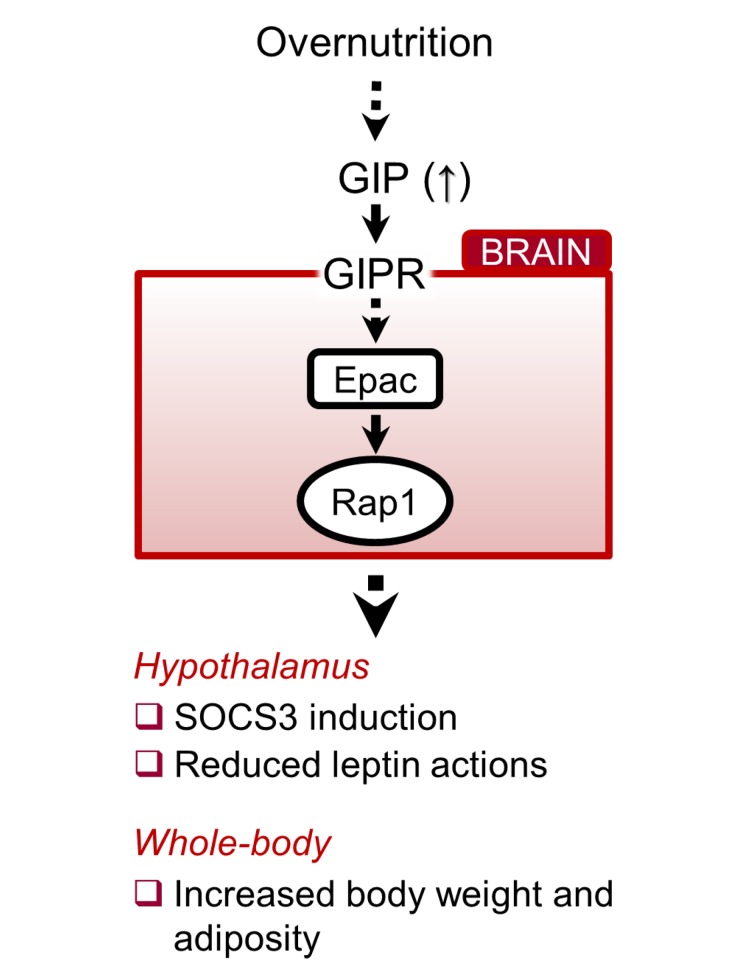

## Introduction

The hypothalamus is a critical site that controls energy balance. Excess calorie intake provokes hypothalamic activation of multiple inflammatory and stress response pathways, such as IKB kinase-β/NF-κB (IKKβ/NF-κB) signaling (1), TLR4 signaling (2), unfolded protein response (UPR) signaling (3), and exchange protein directly activated by cAMP (EPAC)/Rap1 GTPase (EPAC/Rap1) signaling (4). Aberrant activation of these key hypothalamic intrinsic pathways likely impedes neural actions of leptin and central regulation of food intake and body weight, ultimately leading to obesity. Here, we aimed to identify a physiological signal that arises from excess caloric intake and negatively controls hypothalamic leptin action.

The gut-derived hormone glucose-dependent insulinotropic polypeptide, also known as gastric inhibitory polypeptide (GIP), is a well-established incretin hormone (5–8) that directly acts on β cells to stimulate insulin secretion. GIP has also emerged as a critical player in the control of energy balance under conditions of nutrient excess (9). Circulating levels of GIP are elevated during obesity and after consumption of fat or sugar (5–8). Genetic and pharmacological inhibition of GIP and its receptor protects against high-fat diet–induced (HFD-induced) body weight gain (9–14). Furthermore, GWAS have identified GIP receptor (*Gipr*) variants that correlate with obesity (15, 16). Interestingly, both GIPR agonism and antagonism improve body weight in obese animals and humans (17–21). Thus, it is of particular interest to elucidate GIPR sites of action and mechanisms mediating its effects on obesity.

## Results and Discussion

**Figure 1:**
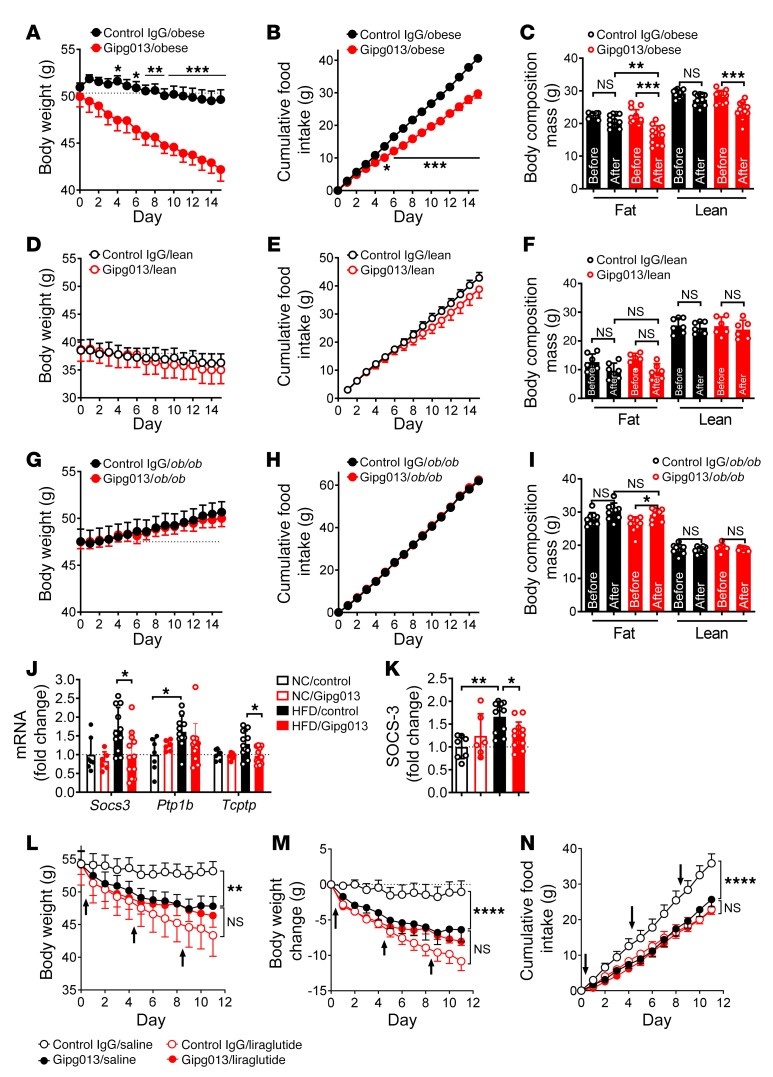
Brain GIPR controls body weight and adiposity in obese mice. The GIPR monoclonal antibody Gipg013 was centrally infused (1 μg, every other day) into HFD-induced obese mice (**A**–**C**, 20 weeks of HFD feeding, *n* = 11–13), normal chow–fed (lean) mice (**D**–**F**, *n* = 6–7), and *ob/ob* mice (**G**–**I**, *n* = 8–9). Body weight (**A**, **D**, and **G**) and food intake (**B**, **E**, and **H**) were measured daily. Body composition (**C**, **F**, and **I**) was measured on day 14 of Gipg013 treatment. (**J**) Relative mRNA expression of the indicated genes in the hypothalamus after 15 days of Gipg013 injection. (**K**) Western blot quantification of SOCS-3 protein in the hypothalamus of Gipg013-treated mice (*n* = 7–13). β-Actin was used as a loading control. (**L**–**N**) HFD-induced obese mice fed for 49 weeks were i.c.v. infused with Gipg013 or control IgG (1 μg every 4 days, arrows) and in combination with an i.p. injection of liraglutide or saline (0.3 mg/kg once a day) (*n* = 9–11). (**L**) Body weight, (**M**) body weight change, and (**N**) food intake were measured during the treatment. Each data point represents the mean ± SEM. *P < 0.05, **P < 0.01, ***P < 0.001, and ****P < 0.0001, by 2-way ANOVA followed by Sidak’s multiple comparisons tests (**A**–**I** and **L**–**N**); 1-way ANOVA followed by Tukey’s multiple comparisons test (**K**); and *t* test (**J**).

First, we confirmed *Gipr* expression in the brain (22) (Supplemental Figure 1, A–C; supplemental material available online with this article; doi:10.1172/JCI126107DS1). To examine the potential role of brain GIPR, we assessed the direct impact of acute inhibition of brain GIPR on obesity by centrally infusing the neutralizing monoclonal antibody Gipg013, which is a highly specific and potent antagonist of GIPR with a fully characterized mode of action (23). Remarkably, central administration (i.c.v.) of Gipg013 significantly reduced the body weight of HFD-induced obese mice (Figure 1A), whereas no effect was observed in mice treated with an isotype control antibody. Food intake (Figure 1B and Supplemental Figure 2A), and fat mass (Figure 1C) were also significantly reduced in Gipg013-treated obese mice. Blood glucose and serum levels of leptin and insulin were decreased in HFD-induced obese mice treated with Gipg013 (Supplemental Figure 2B). The body weight–lowering effect of Gipg013 is probably attributable to reduced food intake, because energy expenditure did not differ between Gipg013- and control IgG-treated obese mice (Supplemental Figure 3). In contrast, in normal chow–fed lean mice, central Gipg013 administration did not reduce body weight, food intake, or fat mass (Figure 1, D–F), indicating that the effects are specific to diet-induced obesity. In agreement with a recent study (21), peripheral administration of Gipg013 did not reduce weight from the baseline but merely prevented weight gain in HFD-induced obese mice (Supplemental Figure 2, C–F). These data collectively indicate a key role of central GIPR signaling in diet-induced obesity. Central administration of Gipg013 into leptin-deficient *ob/ob* mice, another mouse model of obesity, did not induce any improvement in energy balance (Figure 1, G–I), suggesting that Gipg013 in the brain acts through leptin signaling. These central effects of GIPR antagonism are different from those in GIPR deficiency in *ob/ob* mice (9) or obese mice treated peripherally with a GIPR antagonistic antibody (21). The differences might be due to distinct sites of actions of GIPR (e.g., the CNS vs. the periphery). In line with this, brain infusion of Gipg013 significantly decreased expression of the leptin signaling inhibitor *Socs3* (Figure 1, J and K). Although peripheral GIPR antagonism was reported to potentiate a weight-lowering effect of GLP-1 agonists (21), we did not detect an enhanced effect of central Gipg013 and liraglutide on weight loss (Figure 1, L–N), suggesting that GLP-1 is probably not involved in the process.

**Figure 2:**
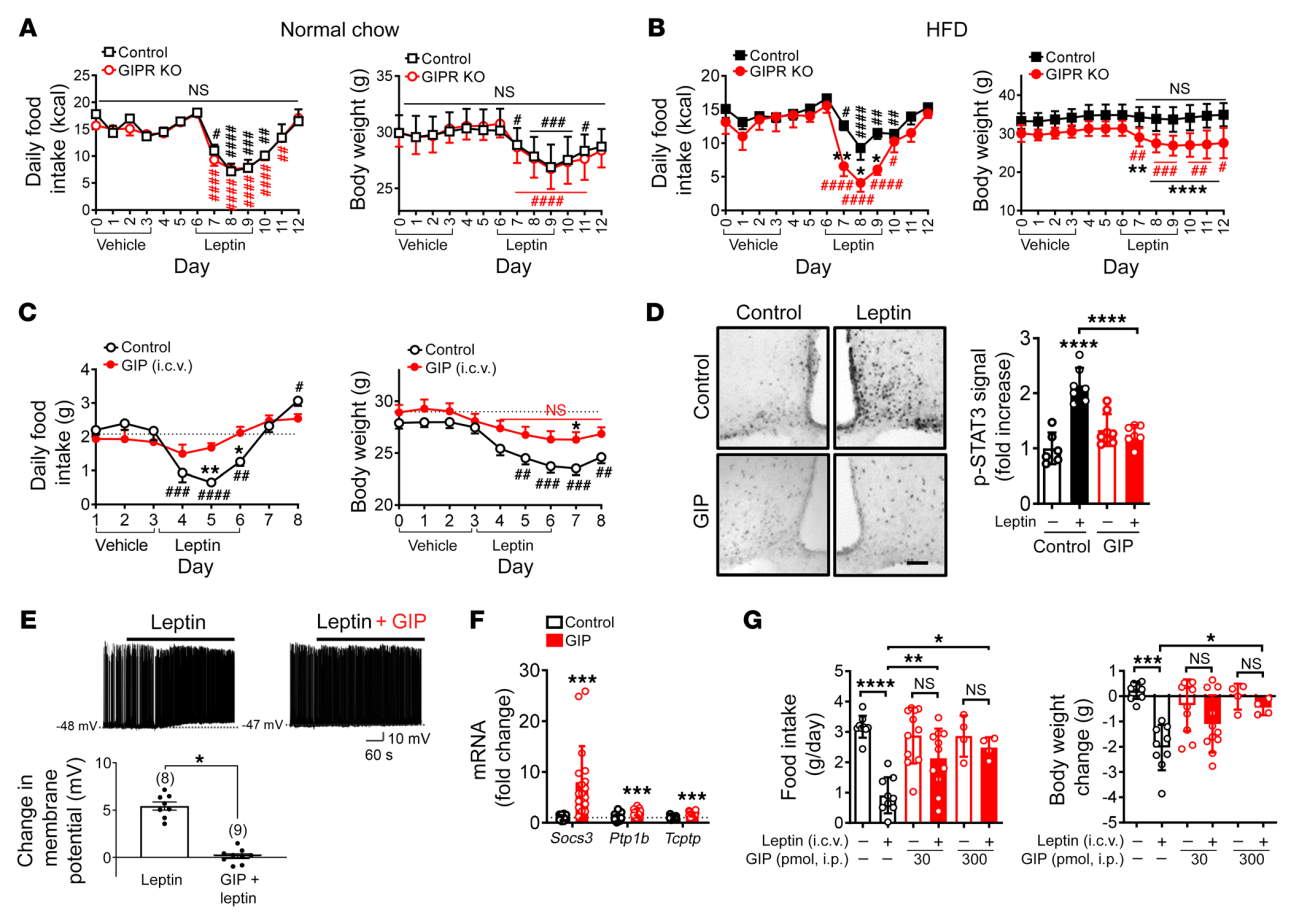
GIP negatively regulates neural leptin actions. (**A** and **B**) Leptin or vehicle was i.c.v. infused into WT and *Gipr*-KO mice after 4 weeks of a normal chow diet (**A**) or a HFD (**B**) (*n* = 7–11). Body weight and food intake were measured daily. (**C**) Normal chow–fed mice (*n* = 11–12, 16 weeks of age) were i.c.v. administered GIP (30 pmol/day) or vehicle. Leptin (5 μg/day) or vehicle was i.c.v. administered. Body weight and food intake were measured. (**D**) Mice (*n* = 3) were i.c.v. administered GIP or vehicle followed by leptin (5 μg) 3 hours later. p-STAT3 immunohistochemistry and quantification. Scale bar: 100 μm. (**E**) Electrophysiological recordings demonstrated that GIP pretreatment (6 h) occluded the leptin-induced depolarization of POMC neurons. The inhibitory effect of GIP on leptin-induced activation of POMC neurons is summarized in the histogram (*n* = 8–9). (**F**) GIP (administered i.c.v.) increased hypothalamic mRNA expression of *Socs-3*, *Ptp1b*, and *Tcptp*. Data are from 3 different experiments (*n* = 17–18). (**G**) Mice received once-daily i.p. injections of GIP for 3 days and then i.c.v. injections of leptin (5 μg) 2 hours after the last GIP injection. Body weight and food intake were measured 24 hours after leptin injection. *n* = 11 for groups without GIP treatment, *n* = 9 for GIP (30 pmol) treatment, and *n* = 4 for GIP (300 pmol) treatment. Each data point represents the mean ± SEM. *P < 0.05, **P < 0.01, ***P < 0.001, and ****P < 0.0001 compared with control mice, by 2-way ANOVA followed by Sidak’s multiple comparisons test (**A**–**D** and **G**); ^#^*P* < 0.05, ^##^*P* < 0.01, ^###^*P* < 0.001, and ^####^*P* < 0.0001, compared with control mice on day 6 (**A** and **B**) and on day 3 (**C**), by 1-way ANOVA followed by Tukey’s multiple comparisons test; and *P < 0.05 and ***P < 0.001 compared with control, by *t* test (**E** and **F**). Data represent the mean ± SEM of 2 different experiments.

Because central inhibition of GIPR resulted in a leptin-dependent antiobesity effect, we investigated the role of GIPR in leptin action in diet-induced obesity by assessing the response of *Gipr*-deficient mice (*Gipr*-KO) (9) and WT mice to exogenously administered leptin. Under normocaloric conditions, central injection of leptin resulted in significantly reduced body weight and suppressed food intake in both *Gipr*-KO and WT mice (Figure 2A). In contrast, under HFD conditions, WT mice did not exhibit these responses to leptin, demonstrating the expected diminished leptin response induced by HFD feeding; *Gipr*-KO mice, however, retained their sensitivity to leptin (Figure 2B). Since age-, body weight–, and adiposity-matched littermates were used as controls (Figure 2B and Supplemental Figure 4A), the observed effect of *Gipr* deficiency on leptin sensitivity was independent of the lean phenotype displayed by *Gipr*-KO mice. Collectively, our data suggest that *Gipr* is necessary for diminished responses to exogenous leptin in diet-induced obese mice.

To directly test whether activation of GIPR in the brain negatively regulates hypothalamic leptin actions, we performed a stereotaxic injection of GIP into the lateral ventricle of lean C57BL/6J mice and assessed central leptin sensitivity. We found that i.c.v. infusion of GIP blunted the anorectic response to exogenous leptin (Figure 2C) as well as leptin-dependent hypothalamic phosphorylation of STAT3 (p-STAT3), a critical mediator of leptin actions (Figure 2D). Importantly, we did not observe these inhibitory effects of GIP in mice lacking *Gipr* (Supplemental Figure 4, B and C), demonstrating that GIP acts through its receptor to blunt leptin-dependent effects. Consistently, GIP increased the hypothalamic levels of *Socs3* (Figure 2F). In addition, GIP pretreatment completely blunted leptin-induced neural activation of pro-opiomelanocortin (POMC) neurons, which are known to mediate leptin-induced anorectic responses, whereas leptin depolarized neurons expressing both POMC and the leptin receptor in control slices (Figure 2E and Supplemental Figure 5). Altogether, these data suggest that GIP drives neuronal leptin resistance.

Since endogenous GIP is produced in K cells in the upper gut and GIP levels are reported to be elevated in diet-induced obesity, reaching 20–100 pM (9, 14, 24, 25), we next determined whether increasing the peripheral levels of GIP inhibits neural leptin actions. We administered GIP through i.p. infusions into lean C57BL/6J mice for 3 days and assessed central leptin sensitivity. Peripheral injection of GIP, at a dose to achieve physiological levels similar to those observed in obese animals (Supplemental Figure 6A), markedly blunted anorectic responses to exogenously administered leptin (Figure 2G). Insulin, leptin, and glucose levels were not significantly altered after 3 days of GIP infusion (Supplemental Figure 6, B and C). Given the growing evidence that peripherally injected GIP can reach the brain (refs. 26–28 and Supplemental Figure 7), these data demonstrate that central effects of leptin are partially blunted by peripheral administration of GIP.

**Figure 3:**
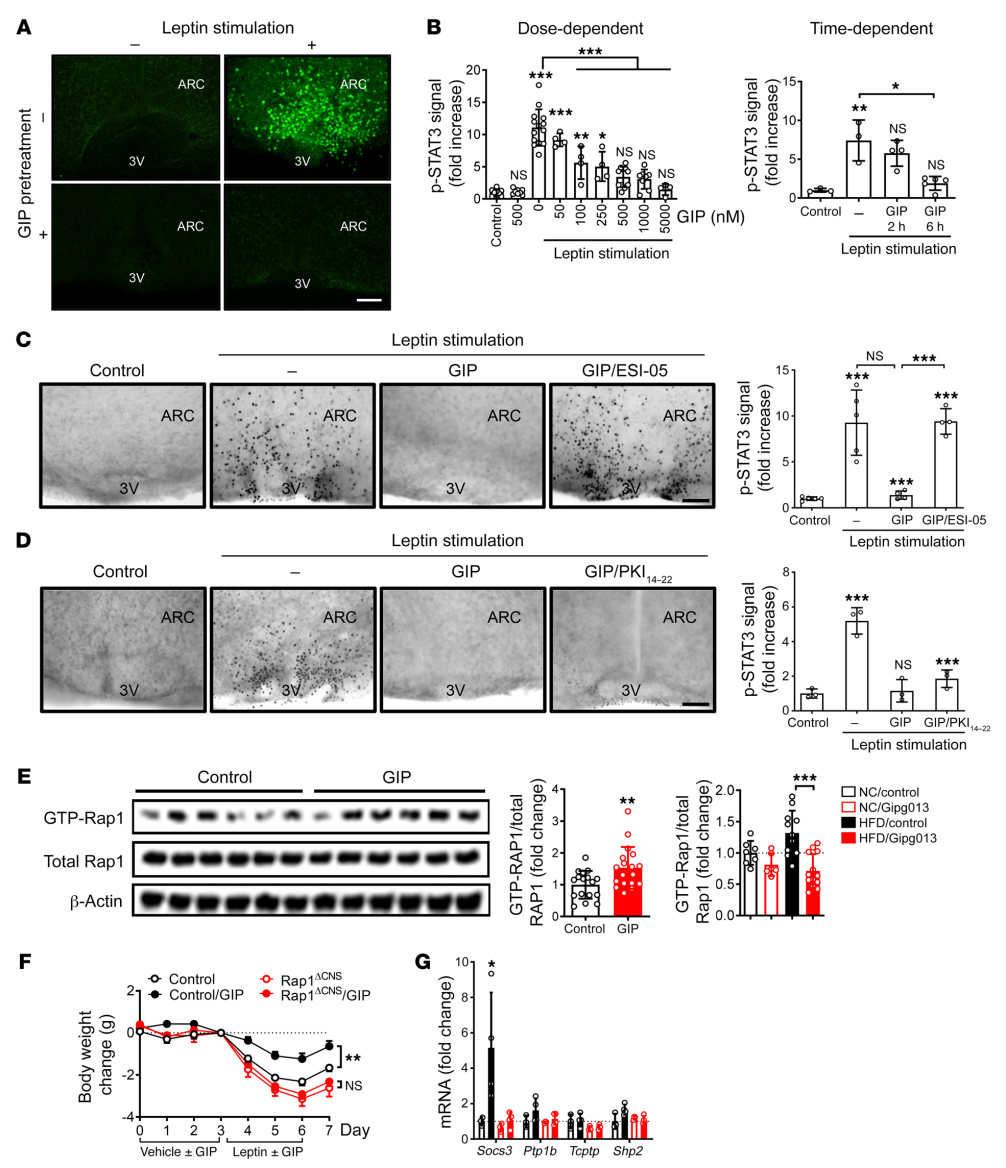
Rap1 mediates the effects of centrally administered GIP. (**A**) Organotypic brain slices were incubated with GIP (0.5 μM, 6 h) and then stimulated with leptin (120 nM, 60 min). Images show p-STAT3 immunostaining of fixed slices. Scale bar: 100 μm. (**B**) GIP inhibited leptin-induced p-STAT3 in a dose- and time-dependent manner (*n* = 3–14). (**C** and **D**) Brain slices were incubated with GIP (0.5 μM), with or without 50 μM ESI-05 (**C**) or 10 μM PKI1_14–22_ (**D**) for 6 hours and then stimulated with 120 nM leptin for 60 minutes. Representative images and quantification of hypothalamic p-STAT3 (*n* = 3–5) are shown. Scale bars: 100 μm. (**E**) Lean mice were i.c.v. administered GIP (3 nmol) for 2 hours. Left: Western blot images of active Rap1, total Rap1, and β-actin (*n* = 6). Middle: Quantification is shown from 3 independent experiments (*n* = 17–18). Right: Graph shows Rap1 activity in the brains of lean and obese mice treated with Gipg013 or control IgG (*n* = 7–10). (**F** and **G**) Rap1^ΔCNS^ or control mice (*n* = 7–9) were i.c.v. injected with GIP (3 nmol/day) or vehicle and then i.c.v. injected with leptin (5 μg/day) 4 hours later. (**F**) Body weight change was measured daily. (**G**) Relative mRNA expression of the indicated genes in the hypothalamus of GIP- or vehicle-treated Rap1^ΔCNS^ mice. Each data point represents the mean ± SEM. *P < 0.05, **P < 0.01, and ***P < 0.001, by 1-way ANOVA followed by Tukey’s multiple comparisons test (**B**–**E** and **G**); **P < 0.01, by *t* test (**E**); and **P < 0.01, by 2-way ANOVA followed by Sidak’s multiple comparisons test (**F**). ARC, arcuate nucleus; 3V, third ventricle.

Next, we sought to identify an intracellular mediator of GIP action in the brain in ex vivo brain slices. Since GIPR couples to cAMP-related signaling (5–8), we examined the involvement of protein kinase A (PKA) and EPAC, two key downstream components of the cAMP pathway. As previously shown (29), leptin robustly induced hypothalamic p-STAT3 levels in brain slices (Figure 3, A and B, and Supplemental Figure 8A). In contrast, leptin-induced hypothalamic p-STAT3 levels were blunted in the slices pretreated with a native GIP peptide in a dose- and time-dependent manner (Figure 3, A and B). An inactive GIP peptide (GIP_3–42_) failed to show an inhibitory effect (Supplemental Figure 8C). GIP also increased SOCS3 protein levels ex vivo (Supplemental Figure 8B). We found that the inhibitory effect of GIP was completely blocked with either ESI-05, an EPAC2-specific inhibitor (Figure 3C), or ESI-09, a specific inhibitor for both EPAC1 and EPAC2 (data not shown), but the inhibitory effect of GIP was not affected by the PKA inhibitor PKI_14–22_ (Figure 3D) or H89 (Supplemental Figure 8D), suggesting that the process is EPAC mediated. Consistently, in ex vivo brain slices, we further observed GIP increases in the amount of the active GTP-bound form of the small GTPase Rap1, which is the direct target of EPAC (Supplemental Figure 8E) or after i.c.v. injection of GIP into lean mice (Figure 3E). In contrast, Gipg013 treatment resulted in a decrease in active Rap1 (Figure 3E). Because neural Rap1 was previously shown to sufficiently drive leptin resistance and be causally related to HFD-induced obesity (4), we reasoned that Rap1 could be a mediator of GIP signaling in the brain. To conclusively test this, we centrally injected GIP into mice with *Rap1* deficiency in the forebrain, including multiple hypothalamic nuclei (Rap1^ΔCNS^) (4, 30), or into control mice. Remarkably, we found that Rap1^ΔCNS^ mice were protected from GIP-mediated leptin resistance and hypothalamic induction of SOCS3 expression, whereas their littermate controls clearly developed GIP-dependent leptin resistance (Figure 3, F and G). Thus, these data indicate that GIP and its receptor are necessary and sufficient for Rap1 activation in the brain and, moreover, that Rap1 activation is required to elicit GIP-induced leptin resistance.

In summary, we have identified a gut-brain axis that involves GIP action on hypothalamic metabolic signaling to drive leptin resistance in obesity. The results suggest that elevated circulating GIP levels in obesity (9, 14, 24, 25) drive both activation of brain Rap1 and neural leptin resistance (Supplemental Figure 9). Our model also reveals what to our knowledge is a unique and previously unidentified molecular pathway linking the GIPR to obesity via EPAC/Rap1 signaling in the brain (Supplemental Figure 9), which further illuminates a functional link between 2 previously unrelated obesity susceptibility genes, *Gipr* (16, 31) and *Rapgef3* (EPAC1) (31).

## Methods

Detailed methods are provided in the Supplemental Methods.

### Study approval.

All procedures for the use of the mice followed protocols approved by the IACUCs of the Baylor College of Medicine and AstraZeneca.

## Author contributions

MF conceived the study. KK, YF, HYL, ELC, KWW, and MF designed the experiments. KK, YF, HYL, ELC, YG, TY, KS, PX, SSC, JN, VH, and MHC performed the experiments. PR contributed reagents and intellectually assisted with studies involving Gipg013. KK, YF, ELC, QM, YG, TY, KS, PX, MHC, YX, KWW, JN, VH, PR, and MF analyzed data and interpreted the results. The majority of the manuscript was written by MF, with some help from KK. All authors approved the final version of the manuscript. The order of the co–first authors was determined by their relative contribution to this study.

## Supplementary Material

Supplemental data

## Version 1 08/12/2019

Electronic publication

## Version 2 09/03/2019

Print issue publication
