# Supplementary material for: Gut-derived GIP activates central Rap1 to impair neural leptin sensitivity during overnutrition
Source: J Clin Invest. 2019 Aug 12;129(9):3786–91. doi: 10.1172/JCI126107 (PMC6715359; doi:10.1172/JCI126107)
Supplement: Supplemental data [file jci-129-126107-s198.pdf]

Supplemental Methods

Mice

Mice were used for all experiments. C57BL/6J mice were obtained from the Jackson Laboratory. Gipr knockout mice (Riken BRC 00782) were provided by Riken BRC (1). Rap1a$^{loxp/loxp}$/Rap1b$^{loxp/loxp}$ mice were provided by Dr. Alexei Morozov (2). Rap1$^{\Delta CNS}$ mice were generated by mating *CaMKII-Cre* mice to Rap1a$^{loxp/loxp}$/Rap1b$^{loxp/loxp}$ mice (3). All mice were maintained on a 12:12 h light-dark cycle condition and temperature-controlled environment with *ad libitum* access to water and normal diet (Pico Lab 5V5R) or high-fat diet (60% kcal fat; Research diet, D12492).

Organotypic brain slice culture

The hypothalamic slices were made as previously described (4).

Immunohistochemistry

Under deep anesthesia, mice were intracardially perfused with saline and 10% formalin. The brains were removed, post-fixed in 10% formalin, and infiltrated with 20% sucrose, and cut into 30 μm slices. The sections were washed 6 times for 5 minutes each in PBS and then for 30 minutes in 0.3% hydrogen peroxide in 0.25% Triton X-100 in PBS (PBT). The sections were then incubated for 48-72 hours with pSTAT3 antibodies (1:3,000, Cell Signaling Technology, 9131) in 3% normal donkey serum with PBT with 0.02% sodium azide. The sections were then reacted with a biotinylated secondary antibody against rabbit IgG (1:1,000, Jackson ImmunoResearch Laboratories, 101097-810, West Grove, PA) followed by the avidin-biotin-peroxidase complex kit (1:1,000, Vectastain Elite ABC kit; Vector Labs, Burlingame, CA). These immunoreactivities were visualized by incubation with 3,3'-diaminobenzidine (DAB, Sigma, St. Louis, MO). After dehydration through graded ethanol, the slides were then immersed in xylene and coverslipped. Images were analyzed using a brightfield Leica microscope. For organotypic slices, the slices that were cut out from the membrane were rinsed 3 times for 10 minutes each in PBS, pH 7.4 and then for 20 min in 1% hydrogen peroxide and 1% sodium hydroxide in PBS to quench endogenous peroxidase activity. Following a series of PBS washes, slices were incubated for 48-72 h at 4 °C in pSTAT3 antibodies (1:3,000) in 3% normal donkey serum (Jackson ImmunoResearch Laboratories) with PBT and 0.02% sodium azide. After washing in PBS, slices were incubated in a biotinylated donkey anti-rabbit antibody (1:1,000) in 3% donkey serum

in PBT for 1 h at room temperature. Tissues were then rinsed in PBS and incubated in ABC (1:500) or streptavidin Alexa Fluor488 conjugate (S-11223, Life Technologies Corporation) for 1 h. For DAB staining, slices were washed in PBS, then reacted in DAB. For immunofluorescence staining, slices were then rinsed in PBS and mounted on slides using Vectashield. To measure pSTAT3 signal in the arcuate nucleus, uneven background was eliminated with Adobe Photoshop and then intensity of staining was measured with NIH Image J.

Total protein extraction and western blot analysis

Proteins were extracted by homogenizing samples in lysis buffer [25 mM Tris-HCl pH 7.4, 150 mM NaCl, 1% NP-40, 1 mM EDTA, 5% glycerol (87787 and 87788 Pierce IP Lysis Buffer, Thermo Fisher Scientific)], with protease and phosphatase inhibitor cocktails (1:100, 78442, Thermo Fisher Scientific). Equal amounts of the samples were separated by SDS-PAGE and transferred to a nitrocellulose membrane by electroblotting. The following primary antibodies were used for western blot assays: anti-phospho-STAT3 (1:1,000, Cell Signaling Technology, 9131), anti-STAT3 (1:1,000, Cell Signaling Technology, 9139), anti-phospho-S6K (1:1,000, Cell Signaling Technology, 9204), anti-S6K (1:1,000, Cell Signaling Technology, 9202), anti-SOCS3 (1:600, Abcam, ab16030), anti-phospho-AKT (1:1,000, Cell Signaling Technology, 4060), anti-AKT (1:1,000, Cell Signaling Technology, 2920), anti-phospho-GSK-3β (1:1,000, Cell Signaling Technology, 9323), anti-GSK-3β (1:1,000, Cell Signaling Technology, 9315), and anti-β-actin (1:10,000, Sigma, A3853). After incubation with primary antibodies for 48-72 h at 4 C°, the membranes were incubated in the secondary antibody conjugated to a fluorescent entity: IRDye 680RD goat anti-rabbit IgG (LI-COR Biosciences, 926-68171) and/or IRDye 800CW goat anti-mouse IgG (LI-COR Biosciences, 827-08364) with gentle agitation for 1 h at room temperature., The Odyssey IR imaging system (LI-COR Biosciences) was used to measure the fluorescent intensity.

Total RNA extraction and quantitative real-time PCR

Tissues were quickly removed and frozen in liquid nitrogen and kept in −80 °C until further processing. Total RNA was extracted using the RNeasy Lipid Tissue Kit (QIAGEN Sciences). cDNA was generated by iScript RT Supermix (Bio-Rad Laboratories) and used with SsoAdvanced Universal SYBR Green Supermix (Bio-Rad Laboratories) for quantitative real-time PCR analysis. qPCR assays were performed using a CFX384 Touch Real-

Time PCR Detection System (Bio-Rad Laboratories). Normalized mRNA levels were expressed in arbitrary units obtained by dividing the averaged, efficiency-corrected values for sample mRNA expression by that for cyclophilin mRNA expression for each sample. The resulting values were expressed as fold change above average control levels. The primer sequences were as follows: SOCS3 (F-CACCTGGACTCCTATGAGAAAGTG and R-GAGCATCATACTGATCCAGGAACT), PTP1B (F-GGAACAGGTACCGAGATGTCA and R-AGTCATTATCTTCCTGATGCAATT), TCPTP (F-AGGGCTTCCTTCTAAGG and R-GTTTCATCTGCTGCACCTTCTGAG), Gipr5/6 (F- GGATCTTGGAGAGACCACAC and R-CTAGCAGCAGAGTCGTCAGG), Gipr (F-AGCTGTGTCAGTGGAGATTG and R-TCCCAAGGCAGAACCAACTC) or Cyclophilin (F-TGGAGAGCACCAAGACAGACA and R-TGCCGGAGTCGACAATGAT).

Electrophysiology

Whole-cell patch-clamp recordings were performed in 17 arcuate Pomc-hrGFP::Lepr-cre::tdtomato neurons from PLT mice as previously described (5, 6).

Detection of GTP-Rap1 by Rap1 pull-down assay

Rap1 pull-down assay was performed using the Active Rap1 Pull-Down and Detection Kit (Thermo Fisher Scientific) as described previously (4).

Cannula implantation and treatments

Mice were anaesthetized with isoflurane and positioned in a stereotaxic frame. The skull was exposed and a 26-gauge single stainless steel guide cannula (C315GS-5-SPC, Plastics One, Roanoke, VA, USA) was implanted into the lateral ventricle (−0.9 mm from the bregma, ±0.5 mm lateral, −2.5 mm from the skull). The cannula was secured to the skull with screws and dental cement. After ICV cannulation, the mice were housed singly and given at least 1 week to recover. On experimental days, the mice were infused with 1 μL of each solution: (1) vehicle (Dulbecco's PBS, dPBS, Sigma-Aldrich, D8537) or GIP (0.03−3 nmol/mouse, Phoenix Pharmaceuticals or Tocris Bioscience), (2) vehicle (dPBS), GIP, leptin (5 μg, Harbor-UCLA Research and Education Institute) or GIP/Leptin, (3) vehicle

(IgG, Sigma-Aldrich, I4506) or Gipg013 (1 μg, MedImmune), (4) vehicle (saline), GIP, Insulin (5 mU, Humulin-R 100 U/ml, Lilly), (5) vehicle (dPBS), GIP, Gipg013 or GIP/Gipg013, (6) vehicle (dPBS) or leptin.

Peripheral injections of Gipg013

Gipg013 and isotype control antibodies were produced in CHO cells by transient transfection as described previously (7). The antibodies were purified using MapSelect SuRe chromatography and concentrated and buffer exchanged using a Millipore Labscale (500 mL) TFF System into 590 mM sodium Acetate, 100 m M NaCl, pH 5.5. Finally, the antibodies were sterile filtered (0.22 μm) and stored at -80C. Gipg0013 contained 98.4% monomer at 21.28 mg/mL and had endotoxin levels below <0.25 EU/mL (<0.01EU/mg) and was tested for activity in a number of *in vitro* potency and affinity assays. All the data published in Ravn et al (8) and Biggs et al (9) were generated with the same batch of Gipg0013. C57Bl6 mice were obtained from Charles River Labs and placed on a 60% calorie from fat diet (D12492, Research Diets) for 12-16 weeks until their body weights reached ~50g. Mice were singly housed with enrichment on a 12:12 light:dark cycle and had free access to food and water. Mice were screened and sorted by body weight for inclusion in all studies. Leptin-deficient *ob/ob* mice (B6.Cg-Lepob/J) were obtained from Jackson Laboratories at 10 weeks old. Mice were housed 3/cage with enrichment on a 12:12 light:dark cycle with free access to an 18% protein diet (Envigo, Teklad # 2918) and water. Mice were screened and sorted by body weight for inclusion in all studies. Mice were dosed with Gipg013 or isotype control subcutaneously every other day for 2 weeks (15 mg/kg). Body weight was recorded daily. All studies were approved of and performed in accordance with local IACUC guidelines for the humane treatment of animals.

Peripheral injections of a GLP-1 receptor agonist liraglutide

After ICV cannulation, the mice were housed singly and given at least 1 week to recover. On experimental days, the mice were infused with 1 μL of each solution: (1) control IgG (1 μg) or (2) Gipg013 (1 μg, MedImmune) once in four days. Mice were dosed with glucagon-like peptide-1(GLP-1) receptor agonist Liraglutide (Selleckchem, S8256) (0.3 mg/kg) or saline intraperitoneal injection every day. Body weight and food intake were recorded daily.

Cerebrospinal fluid collection and GIP detection

Two hours after intraperitoneal administration of GIP (300 pmol/mouse), cerebrospinal fluid (CSF) was isolated from the fourth ventricle using the cisterna magna puncture method (10). GIP concentrations in CSF were evaluated using the Milliplex mouse metabolic hormone panel kit (MMHMAG-44K, Millipore, Billerica, MA, USA) with MAGPIX instrument according to the manufacturer's instructions.

Leptin sensitivity test

After ICV cannulation, mice were singly housed and acclimatized for 1 week before the study. Age- and body weight-matched cohorts were used. For leptin sensitivity test, mice were administered GIP or vehicle followed by leptin (5 µg, ICV) or vehicle 4 h later. Body weight and cumulative food intake were measured 24 h after the leptin injection.

Body composition and energy expenditure measurements

Measurements of (body composition / energy balance / food intake) were performed in the Mouse Metabolic Research Unit at the USDA/ARS Children's Nutrition Research Center, Baylor College of Medicine (www.bcm.edu/cnrc/mmru). The whole-body composition was measured using NMR imaging (EchoMRI). Body weight- and body composition-matched HFD-induced obese mice (20 weeks of HFD feeding) were used for metabolic assessment. After ICV cannulation, mice were singly housed and acclimatized for 1 week before the study. Mice were then acclimatized to the metabolic cages for 3 days before measurements were taken. During acclimation periods, mice were centrally injected with the vehicle (IgG, 1 µg, every other day). During energy expenditure measurements, mice were ICV-injected with the IgG or Gipg013 (1 µg, every other day). Metabolic parameters, including O_2 consumption, CO_2 production, respiratory exchange ratio (RER), heat production, ambulatory activity and food intake, were determined by using the Columbus Instruments Comprehensive Lab Animal Monitoring System (CLAMS).

Statistics

The data are presented as mean ± SEM. Statistical analyses were performed using GraphPad Prism for a two-tailed unpaired Student's t-test, or One- or Two-way ANOVA followed by post hoc Tukey's, or Sidak's tests. $P < 0.05$ was considered to be statistically significant. Sample sizes were designed to be sufficient to enable statistical

significance to be accurately determined. No samples or animals were excluded from analysis, with the exception of exclusions due to technical errors. For *in vivo* studies, mice were randomly assigned to groups. The investigators were not blinded in the studies. Appropriate statistical analyses were applied, assuming a normal sample distribution, as specified in the figure legends. No estimate of variance was made between each group.

Study approval

All procedures to maintain and use the mice followed protocols approved by the Animal Care and Use Committee at the Baylor College of Medicine (Houston, US) and/or AstraZeneca (Cambridge, UK).

Supplemental Figures

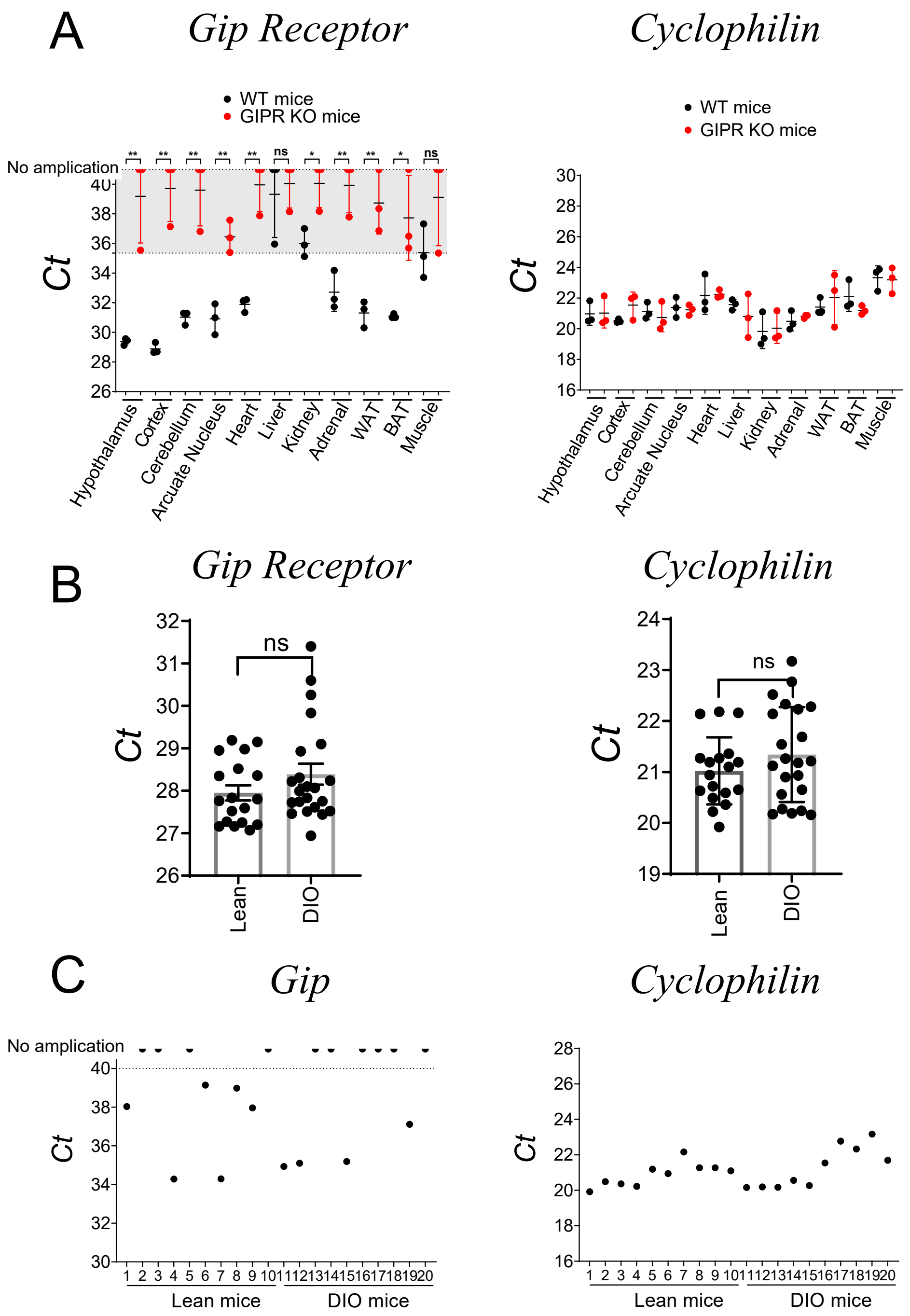

Supplemental Figure 1. GIPR is expressed by the brain. (**A**) Raw *Ct* values of *Gipr* and *cyclophilin* RNAs in RNA samples isolated from the indicated tissues of control mice and *Gipr* KO mice (n=3). *Gipr* mRNA was expressed in the CNS at low levels (29 < Ct < 34) but comparable to that in fat known to produce *Gipr*. Notably, the low signals throughout the CNS are the true signal from the *Gipr* gene because PCR amplification signals were not detected in the tissues of the *Gipr* KO mice. *Ct* values >35 were interpreted as the absence of gene expression because the background *Ct* values were detected at or above 35.5 in the *Gipr* null mice. qPCR primers (Gipr5/6QF and Gipr5/6QR) were designed for targeted exons of *Gipr* KO. (**B**) Raw *Ct* values of *Gipr* and *cyclophilin* RNAs in the hypothalamus of mice fed on either normal chow or a HFD for over 12 months (n=18-21). (**C**) Raw *Ct* values of *Gip* and *cyclophilin* RNAs in the hypothalamus of mice fed on either lean normal chow or a high-fat diet for over 12 months. *P<0.05, **P<0.01, and ***P<0.001 compared to WT, based on t-tests.

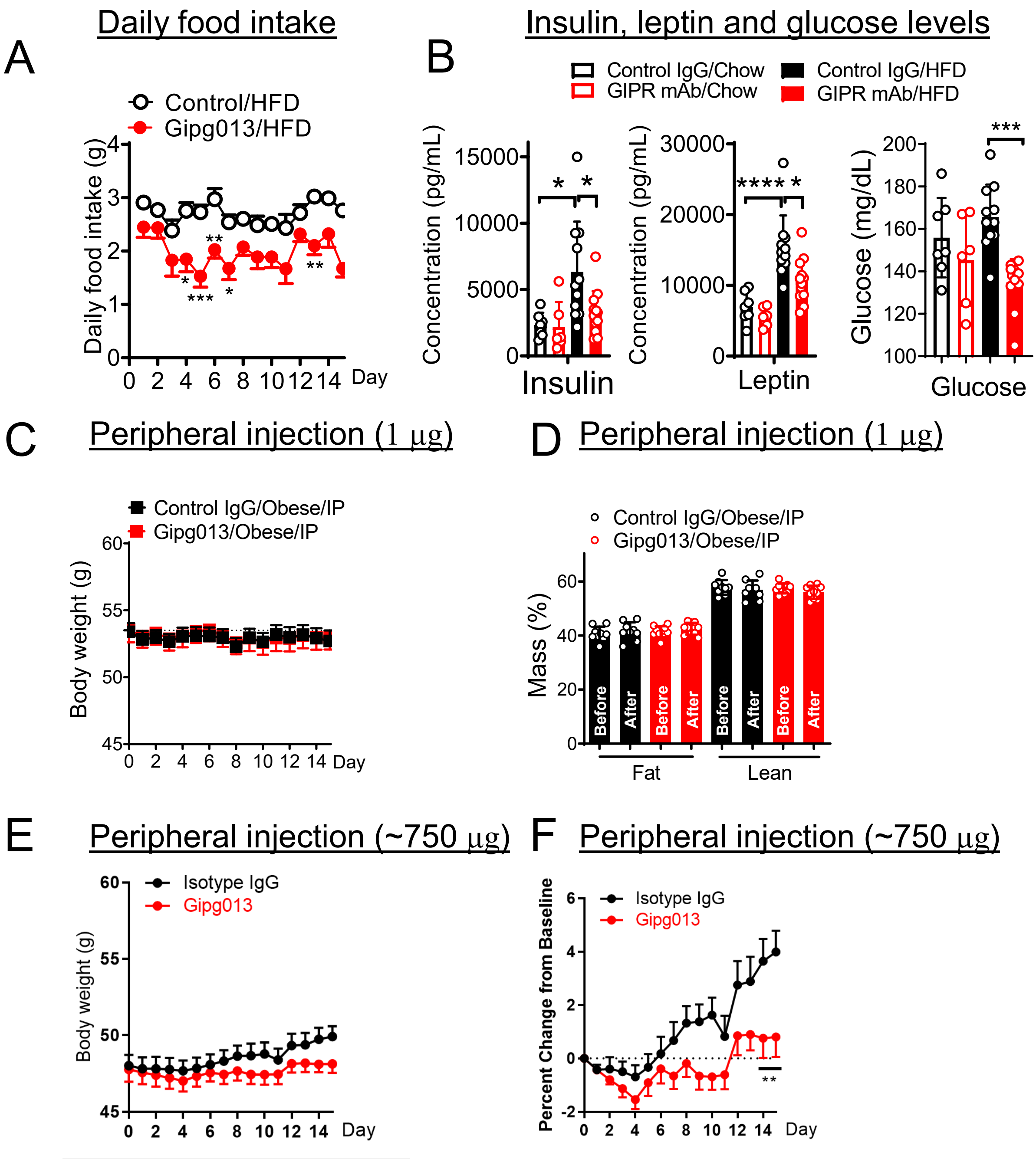

Supplemental Figure 2. The effect of Gipg013 on body weight and food intake. (**A**) Daily food consumption of the same mice receiving Gipg013 or vehicle described in Fig. 1A-C. (**B**) Serum insulin and leptin levels (*Left*) and blood glucose (*Right*) after 15 days of obese mice with Gipg013 injection. (**C** and **D**) Peripherally injected Gipg013 (1 μg) failed to reduce body weight (**C**) and adiposity (**D**) of HFD-obese mice (20 weeks of HFD feeding) (n=9-10). (**E** and **F**) A high dose of Gipg013 (S.C., every other day, 15 mg/Kg corresponding 750 μg per mouse) did not reduce body weight (**E**) but prevented body weight gain (F) of diet-induced obese mice when peripherally administrated. Values are presented as mean ± S.E.M. *p<0.05, **p<0.01, and ***P<0.001 for repeated measures two-way ANOVA with Sidak's multiple comparisons tests.

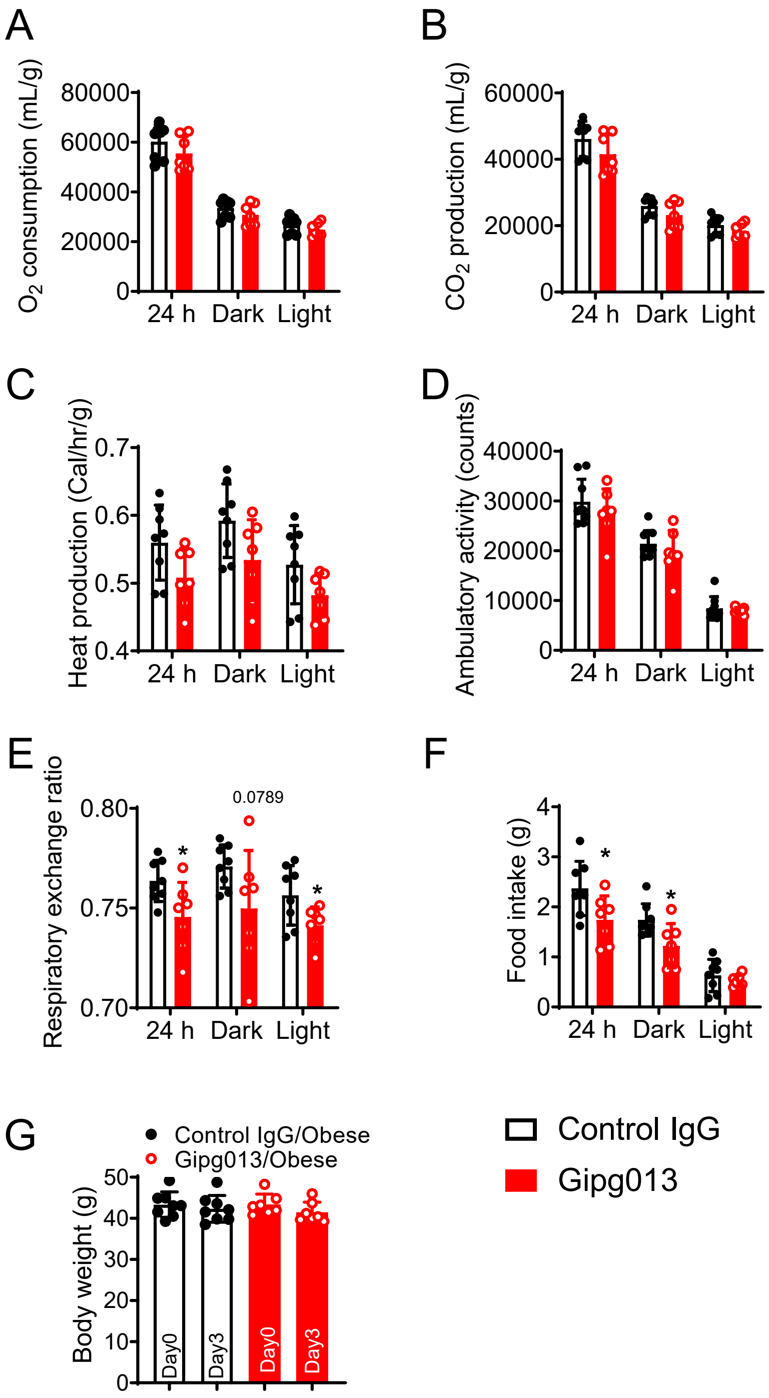

Supplemental Figure 3. Metabolic profile of HFD-fed male mice with a 3-day central injection of the GIPR antibody (Gipg013). Shown are O_2 consumption (**A**), CO_2 production (**B**), heat production (**C**), ambulatory activity (**D**), respiratory exchange ratio (**E**) and food intake (**F**) during 24 hour, dark, or light cycles, and (**G**) body weight at the time of the CLAMS study. n=8-7. *P<0.05 compared to control, based on t-tests.

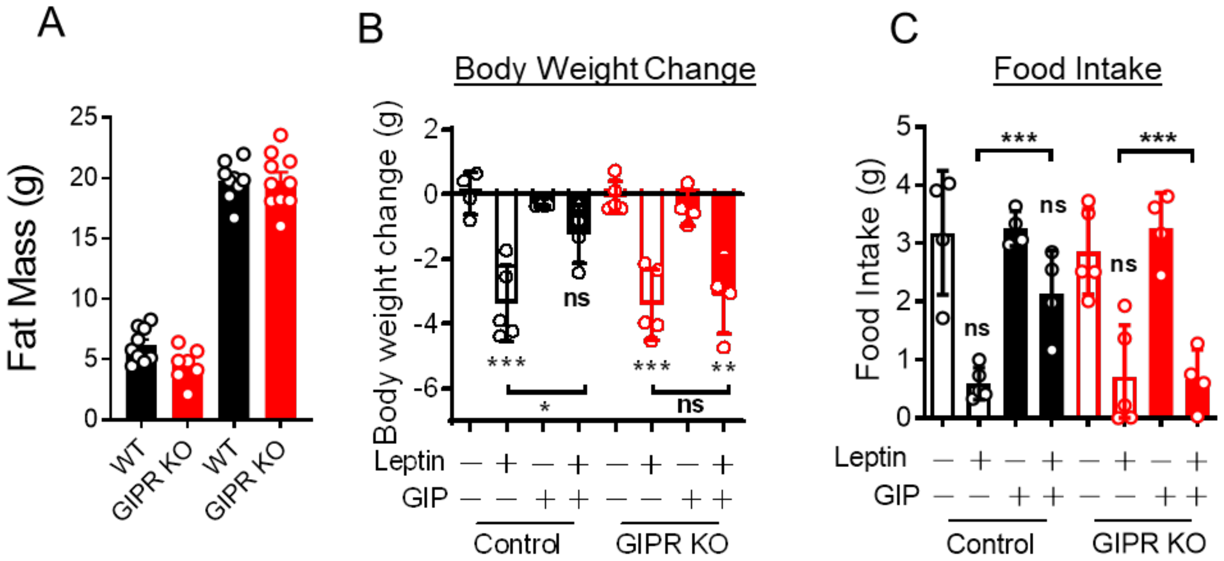

Supplemental Figure 4. GIP partially diminishes the central actions of leptin on food intake and body weight. (**A**) Body composition of *Gipr* KO and their control littermates fed either a chow or a HFD for 4 weeks (the same cohort used in Fig 2 A and B) is shown (n=7-11/group). (**B** and **C**) Centrally administered GIP significantly blunted central action of leptin in control mice but not in *Gipr* KO mice. Mice received ICV injections of GIP (3 nmol) or vehicle (1 µL), and then received a second ICV injection of leptin (5 µg) or vehicle 4 hours later. Body weight (**B**) and cumulative food intake (**C**) were measured 24 hours after the leptin injection (n=4-5 per group). Values are presented as mean ± S.E.M. *p<0.05, **p<0.01, ***p<0.001 for One-way ANOVA followed by Tukey's Multiple Comparison Test.

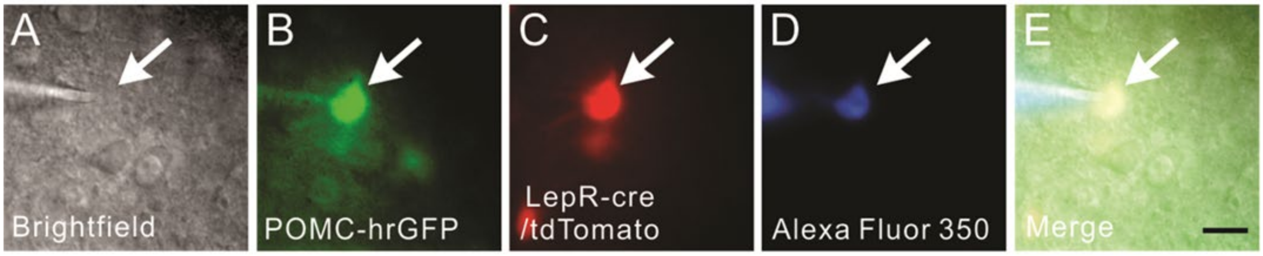

Supplemental Figure 5. Representative images of Pomc-hrGFP::Lepr-cre::tdtomato neuron. (A) Brightfield illumination of Pomc-hrGFP::Lepr-cre::tdtomato neuron from PLT mice. (**B and C)** The same neuron under FITC (hrGFP) and Alexa Fluor 594 (tdtomato) illumination. (**D)** Complete dialysis of Alexa Fluor 350 from the intracellular pipette. (**E)** Merged image of the targeted Pomc neuron. Arrow indicates the targeted cell. Scale bar, 50 µm.

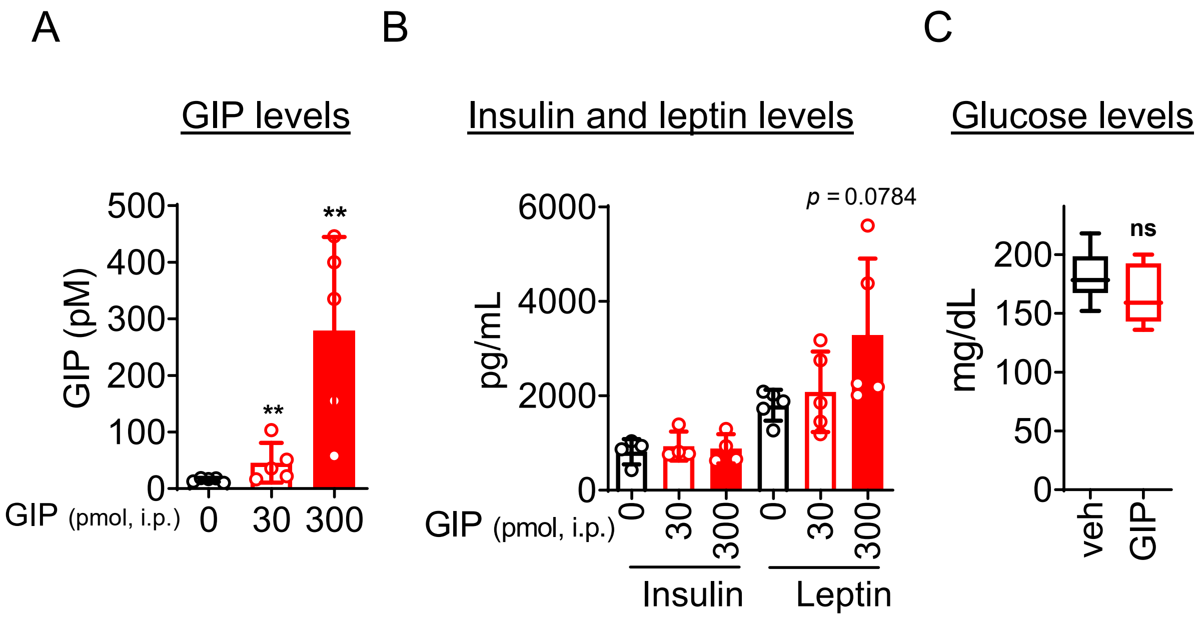

Supplemental Figure 6. GIP, leptin, insulin, and blood glucose levels in mice receiving an infusion of GIP. (**A**) Serum GIP levels in mice receiving ip GIP infusion (IP, 30 or 300 pmol, twice a day for 3 days). Blood was collected two hours after the last injection of GIP (ip), and GIP levels were measured using an ELISA assay for GIP. (**B** and **C**) Serum insulin and leptin levels (**A**) and blood glucose (**B**) after 3 days of lean mice with peripheral injections of GIP (300 pmol for **C**). Blood was collected two hours after the last GIP injection. Values are presented as mean ± S.E.M. *p<0.05, **p<0.01, ***p<0.001 for One-way ANOVA followed by Tukey's Multiple Comparison Test.

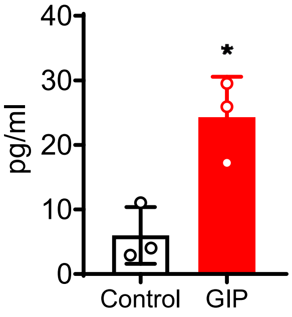

Supplemental Figure 7. Detection of peripherally administered GIP in the cerebrospinal fluid. GIP_{1-42} was injected into C57BL/6 mice (IP, 300 pmol, 2 hours, n = 3). The cerebrospinal fluid was collected through from the cisterna magna of the mice. CSF GIP levels were measured using a Milliplex mouse metabolic hormone panel kit. *p<0.05 for t-test.

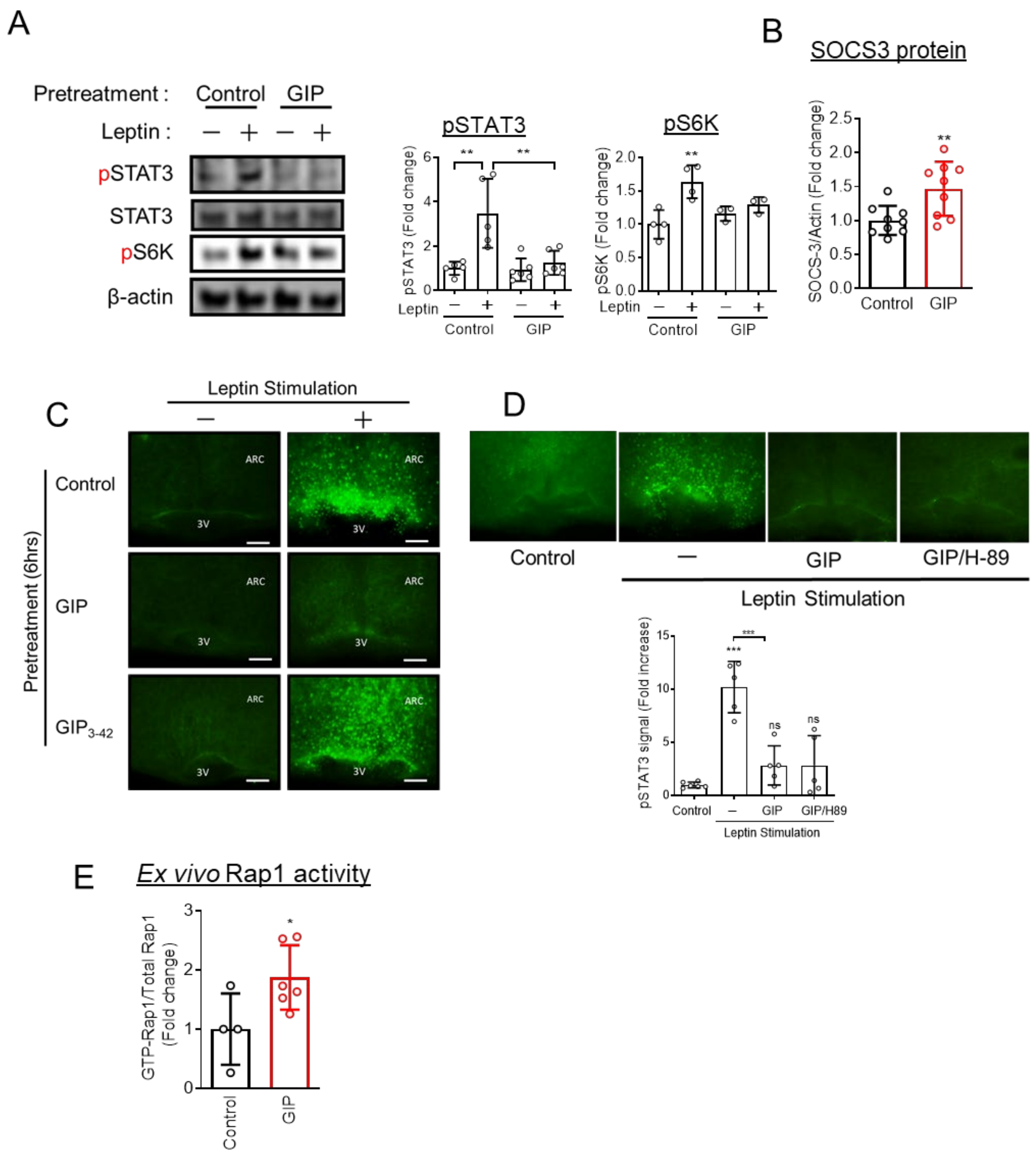

Supplemental Figure 8. Inhibitory effects of GIP on neural leptin action *ex vivo*. (**A**) A representative western blot for pSTAT3 and pS6K in the hypothalamus (*Left*), and its quantification (*Right*, n=3-6), illustrate the inhibitory effects of GIP on leptin signaling. (**B**) Quantification of western blot signal for SOCS3 in the hypothalamus treated with GIP (500 nM) or saline (n=9). (**C**) Organotypic brain slices were incubated with GIP ($GIP_{1\text{-}42}$, 0.5 µM) or inactive GIP peptide ($GIP_{3\text{-}42}$, 0.5 µM) for 6 hours and then stimulated with leptin. Images show pSTAT3 immunostaining of fixed slices. Scale bar, 100 µm. ARC, arcuate nucleus; 3V, third ventricle. Values are presented as mean ± S.E.M. (**D**) Organotypic brain slices were incubated with GIP (0.5 µM) with or without H-89 (10 µM) for 6 hours and then stimulated with leptin (120 nM, 60 min). (*Upper*). Images show pSTAT3 immunostaining of fixed slices. (*Lower*) Quantification of hypothalamic pSTAT3 (n=5-6) in organotypic brain slices. (**E**) GIP increases the GTP-bound, active form of Rap1. Slices (n=4-6) were treated with GIP (500 nM) or saline for 2 hours and then proteins were extracted and Rap1 activity was measured as described in Materials and methods. *p<0.05 and **p<0.01 for Two-way ANOVA followed by Tukey's Multiple Comparison Test in (**A**). *p<0.05 for t-test in (**B** and **E**). **p<0.01 for One-way ANOVA followed by Tukey's Multiple Comparison Test in (**D**)

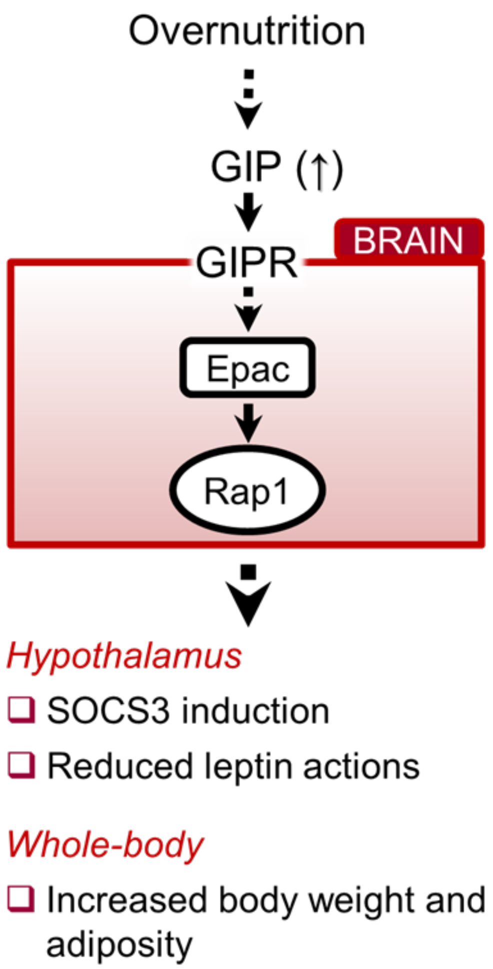

Supplemental Figure 9. Schematic representation of central GIP actions via Rap1. Serum GIP levels are increased in obesity and/or following HFD feeding (11-16). The increased GIP acts on hypothalamic neurons, increases SOCS3 and reduces neural leptin actions, thereby mediating dietary obesity. GIP, Glucose-dependent insulinotropic polypeptide (or gastric inhibitory polypeptide); Epac, the exchange factor directly activated by cAMP; SOCS3, suppressor of cytokine signaling3.

References

1. Miyawaki K, Yamada Y, Yano H, Niwa H, Ban N, Ihara Y, et al. Glucose intolerance caused by a defect in the entero-insular axis: a study in gastric inhibitory polypeptide receptor knockout mice. *Proc Natl Acad Sci U S A.* 1999;96(26):14843-7.
2. Pan BX, Vautier F, Ito W, Bolshakov VY, and Morozov A. Enhanced cortico-amygdala efficacy and suppressed fear in absence of Rap1. *J Neurosci.* 2008;28(9):2089-98.
3. Kaneko K, Xu P, Cordonier EL, Chen SS, Ng A, Xu Y, et al. Neuronal Rap1 Regulates Energy Balance, Glucose Homeostasis, and Leptin Actions. *Cell reports.* 2016;16(11):3003-15.
4. Fukuda M, Williams Kevin W, Gautron L, and Elmquist Joel K. Induction of Leptin Resistance by Activation of cAMP-Epac Signaling. *Cell metabolism.* 2011;13(3):331-9.
5. Cowley MA, Smart JL, Rubinstein M, Cerdan MG, Diano S, Horvath TL, et al. Leptin activates anorexigenic POMC neurons through a neural network in the arcuate nucleus. *Nature.* 2001;411(6836):480-4.
6. Williams KW, Liu T, Kong X, Fukuda M, Deng Y, Berglund ED, et al. Xbp1s in Pomc Neurons Connects ER Stress with Energy Balance and Glucose Homeostasis. *Cell Metab.* 2014;20(3):471-82.
7. Daramola O, Stevenson J, Dean G, Hatton D, Pettman G, Holmes W, et al. A high-yielding CHO transient system: coexpression of genes encoding EBNA-1 and GS enhances transient protein expression. *Biotechnology progress.* 2014;30(1):132-41.
8. Ravn P, Madhurantakam C, Kunze S, Matthews E, Priest C, O'Brien S, et al. Structural and pharmacological characterization of novel potent and selective monoclonal antibody antagonists of glucose-dependent insulinotropic polypeptide receptor. *J Biol Chem.* 2013;288(27):19760-72.
9. Biggs EK, Liang L, Naylor J, Madalli S, Collier R, Coghlan MP, et al. Development and characterisation of a novel glucagon like peptide-1 receptor antibody. *Diabetologia.* 2018;61(3):711-21.
10. Liu L, and Duff K. A technique for serial collection of cerebrospinal fluid from the cisterna magna in mouse. *Journal of visualized experiments : JoVE.* 2008(21).
11. Ahlqvist E, Osmark P, Kuulasmaa T, Pilgaard K, Omar B, Brons C, et al. Link between GIP and osteopontin in adipose tissue and insulin resistance. *Diabetes.* 2013;62(6):2088-94.
12. Bailey CJ, Flatt PR, Kwasowski P, Powell CJ, and Marks V. Immunoreactive gastric inhibitory polypeptide and K cell hyperplasia in obese hyperglycaemic (ob/ob) mice fed high fat and high carbohydrate cafeteria diets. *Acta endocrinologica.* 1986;112(2):224-9.
13. Brons C, Jensen CB, Storgaard H, Hiscock NJ, White A, Appel JS, et al. Impact of short-term high-fat feeding on glucose and insulin metabolism in young healthy men. *The Journal of physiology.* 2009;587(Pt 10):2387-97.
14. Creutzfeldt W, Ebert R, Willms B, Frerichs H, and Brown JC. Gastric inhibitory polypeptide (GIP) and insulin in obesity: increased response to stimulation and defective feedback control of serum levels. *Diabetologia.* 1978;14(1):15-24.
15. Flatt PR, Bailey CJ, Kwasowski P, Swanston-Flatt SK, and Marks V. Abnormalities of GIP in spontaneous syndromes of obesity and diabetes in mice. *Diabetes.* 1983;32(5):433-5.
16. Ponter AA, Salter DN, Morgan LM, and Flatt PR. The effect of energy source and feeding level on the hormones of the entero-insular axis and plasma glucose in the growing pig. *The British journal of nutrition.* 1991;66(2):187-97.
